# Mesenchymal stem cell-mediated immunomodulation of recruited mononuclear phagocytes during acute lung injury: a high-dimensional analysis study

**DOI:** 10.7150/thno.52514

**Published:** 2021-01-01

**Authors:** Jingqi Liu, Pan Li, Jiaqi Zhu, Feiyan Lin, Jiahang Zhou, Bing Feng, Xinyu Sheng, Xiaowei Shi, Qiaoling Pan, Jiong Yu, Jianqing Gao, Lanjuan Li, Hongcui Cao

**Affiliations:** 1 State Key Laboratory for the Diagnosis and Treatment of Infectious Diseases, Collaborative Innovation Center for Diagnosis and Treatment of Infectious Diseases, The First Affiliated Hospital, Zhejiang University School of Medicine, 79 Qingchun Rd., Hangzhou City 310003, China; 2 National Clinical Research Center for Infectious Diseases, 79 Qingchun Rd., Hangzhou City 310003, China; 3 Zhejiang Provincial Key Laboratory for Diagnosis and Treatment of Aging and Physic-chemical Injury Diseases, 79 Qingchun Rd, Hangzhou City 310003, China.; 4 College of Pharmaceutical Sciences and Dr. Li Dak Sum & Yip Yio Chin Center for Stem Cell and Regenerative Medicine, Zhejiang University, Hangzhou 310058, China

**Keywords:** Acute lung injury, Recruited mononuclear phagocytes, Mass cytometry, Single-cell RNA sequencing, Mesenchymal stem cells

## Abstract

**Rationale:** Acute lung injury (ALI)-recruited mononuclear phagocytes play a pivotal role in lung injury and repair. This study investigated the types of recruited mononuclear phagocytes and the immunotherapeutic effects of allograft mesenchymal stem cells (MSCs) in a mouse model of lipopolysaccharide (LPS)-induced ALI.

**Methods:** C57BL/6 mice were orotracheally instilled with LPS (20 mg/kg). Compact bone-derived MSCs were administered orotracheally 4 h after LPS inhalation. Mononuclear phagocytes recruited in the lung tissues were characterized at different timepoints by high-dimensional analysis including flow cytometry, mass cytometry, and single-cell RNA sequencing.

**Results:** Eight mononuclear phagocyte subsets recruited to LPS-challenged lungs were precisely identified. On day 3 after LPS administration, both Ly6C^hi^CD38^+^ and Ly6C^low^CD38^+^ monocytes were recruited into acutely injured lungs, which was associated with increased secretion of neutrophil chemokines. Ly6C^hi^CD38^+^ monocytes differentiated into M1 macrophages on day 3, and subsequently differentiated into CD38^+^ monocyte-derived dendritic cells (mo-DCs) on day 7, while Ly6C^low^CD38^+^ monocytes differentiated into CD11b^+^CD38^+^ DCs on day 7. When ALI mice were treated with MSCs, the mortality significantly reduced. Notably, MSCs reduced the amount of M1 macrophages and reduced the secretion of neutrophil chemokines on day 3. Furthermore, MSCs reduced the number of CD38^+^ mo-DCs and CD11b^+^CD38^+^ DCs on day 7, suppressing the antigen presentation process. Recruited mononuclear phagocyte subsets with a high level of CD38 exhibited an activated phenotype and could secrete higher levels of cytokines and chemokines.

**Conclusions:** This study characterized the dynamic functions and phenotypes of recruited mononuclear phagocytes in ALI mice and MSC-treated ALI mice.

## Introduction

Sepsis-induced acute lung injury (ALI) is an important cause of acute respiratory distress syndrome, which is a life-threatening medical condition with high morbidity and mortality 1. Neutrophil recruitment leading to lung injury is the hallmark of ALI 1, 2. Neutrophils are recruited from the circulation in response to released chemokines, proteases, eicosanoids, and growth factors. Excessive activation of neutrophils with the release of cytotoxic and immune cell-activating cytokines leads to lung damage. Along with neutrophils, monocytes are recruited into injured lungs, where they generate various chemokines to promote neutrophil recruitment and amplify lung injury 3-5. Recruited monocytes in lungs ultimately differentiate into dendritic cells (DCs) or macrophages that regulate tissue repair 6-8.

Monocytes, DCs, and macrophages are regarded as mononuclear phagocytes (MNPs), which play a major role in lung tissue injury and repair 6, 9. Lung MNPs consist of various subsets, including plasmacytoid DCs (pDCs), CD103^+^ and CD11b^+^ DCs, interstitial macrophages, alveolar macrophages (AMs), and monocytes. However, it is challenging to identify cell populations solely on the basis of surface marker expression or functional specialization, because markers are shared among different cell populations and their functional capacities can be acquired or lost during inflammation. For example, monocytes can differentiate into monocyte-derived DCs (mo-DCs) or monocyte-derived macrophages depending on the local inflammation microenvironment. Thus, mo-DCs and monocyte-derived macrophages are difficult to distinguish because of their shared origin and marker expression 10. Furthermore, the characteristics initially used to define recruited MNPs have been shown to define resident cell populations. For example, resident macrophages were initially presumed to undergo renewal by means of recruited monocytes or macrophages, but have since been shown to undergo renewal through self-replication 11, 12.

Although therapies to prevent or treat ALI in sepsis remain elusive, mesenchymal stem cells (MSCs) which are adult stem cells and can differentiate into many somatic cell types (e.g., osteoblasts, chondrocytes, adipocytes, and lung alveolar cells) offer potential promise. Importantly, MSCs can regulate immune responses to promote injured tissue repair, and have thus emerged as potentially attractive candidates for the treatment of ALI 13-15. Previous studies have suggested that MSCs protect against ALI by reducing inflammatory cytokine secretion from monocytes 16, enhancing phagocytosis by macrophage/monocytes 16, or promoting M2 macrophage polarization and enhanced interleukin (IL)-10 secretion 15, 17.

However, the protective role of MSCs through regulation of recruited MNPs in lung tissues remains largely unexplored, and it could be beneficial to explore the MSC-based treatment for ALI. To clarify how MSCs modulate the distribution, function, and differentiation of recruited MNPs, in this study, we performed a high-resolution analysis of heterogeneity and phenotypic diversity of recruited MNPs with MSC treatment in a mouse model of lipopolysaccharide (LPS)-induced ALI, using both mass cytometry 18-20 and single-cell RNA sequencing (scRNA-seq) 21, 22.

To the best of our knowledge, this is the first large-scale report of changes in recruited MNPs during ALI progression and MSC cell therapy. The results indicate that distinct MNP subsets can be found within ALI mouse lung tissues following MSC treatment, and that these subsets exhibit intrinsic plasticity. These findings support the notion that the MNPs recruited during MSC cell therapy in the ALI model consist of multiple heterogeneous cell populations that retain intrinsic plasticity. The phenotypic and functional characterization of the different MNP populations may help to explain the reduced proportions of subpopulations with pro-inflammatory functions, thus providing new avenues for more effective immune-based cell therapy for ALI.

## Methods

### Generation of mouse MSCs

MSCs were isolated from compact bones of C57BL/6 mice. Their isolation, culture, osteogenic and adipogenic induction protocols, and surface marker expression analyses were performed as previously described 23, 24. MSCs at passage 3 were used in all experiments.

### Mouse model of ALI

C57BL/6 mice (6-8 weeks old) were purchased from Nanjing Biomedical Research Institute of Nanjing University. All mice were maintained under specific pathogen-free conditions. All animal experiments were approved by the Ethics Committee of The First Affiliated Hospital of Zhejiang University School of Medicine.

C57BL/6 mice were anesthetized and administered LPS (20 mg/kg) (*Escherichia coli* 0111:B4, Sigma-Aldrich, Poole, UK) orotracheally in phosphate-buffered saline (PBS) to induce ALI.

Mice were randomly assigned to one of the following treatments: PBS (n = 10, administered two doses of 20 μL PBS orotracheally, 4 h apart); LPS (n = 30, administered 20 mg/kg LPS orotracheally to establish the ALI model, then 20 μL of PBS orotracheally 4 h later); or LPS/MSC (n = 30, 4 h after LPS administration, 5 × 10^5^ MSCs were administered orotracheally in 20 μL PBS). Days 3 and 7 after LPS administration were selected for analysis of MSC-related retrieval and tissue analysis. After MSC treatment, mouse survival was recorded at 24-h intervals until day 11 after LPS administration (n = 10).

### Lung histological analysis

Lung specimens of euthanized mice were harvested and fixed in 4% paraformaldehyde and then embedded in paraffin. Specimens were then sectioned at 5-μm thickness and stained with hematoxylin and eosin. Stained sections were imaged using a Nanozoomer 2.0-RS scanner (Hamamatsu, Japan). The severity of lung injury was evaluated based on the alveolar structure and inflammatory cell infiltration.

### Cytokine analysis

Bronchoalveolar lavage fluid (BALF) was collected on days 3 and 7 and cytokine contents were compared among groups. IL-4, IL-6, IL-2, IL-13, IL-10, tumor necrosis factor alpha (TNF-α), transforming growth factor beta (TGF-β), and interferon-gamma (IFN-γ) protein levels were detected using the bead-based Legendplex^TM^ assay (Multi-Analyte Flow Assay Kit, BioLegend, San Diego, CA, USA), in accordance with the manufacturer's instructions. Data were collected on a Cytoflex S flow cytometer (Beckman Coulter, CA, USA) and analyzed using Legendplex V 8.0 software (BioLegend).

### Preparation of pulmonary immune cell suspensions

Lungs of anesthetized mice were perfused with 10 mL PBS through the right ventricle. Whole lungs were then sectioned into small pieces, transferred into gentle MACS C tubes (Miltenyi Biotec, Bergisch Gladbach, Germany) containing enzyme mix (Mouse Lung Dissociation Kit, Miltenyi Biotec), homogenized using a GentleMACS™ Dissociator (Miltenyi Biotec), and digested at 37 ℃ for 30 min. Digested lung tissue homogenates were filtered through a 70-μM MACS SmartStrainer (Miltenyi Biotec), and then subjected to density gradient centrifugation. Red blood cells were then lysed using AKC lysis buffer (Gibco®, Grand Island, NY, USA) to yield single-cell suspensions of pulmonary immune cells.

### Flow cytometry analysis

Antibodies used for staining pulmonary MNPs included BV711 anti-mouse CD45 (30-F11, BioLegend), APC anti-mouse CD11b (M1/70, BD Biosciences, San Jose, CA, USA), PE/Cyanine7 anti-mouse CD11c (N418, BioLegend), AF700 anti-mouse Ly6G (1A8, BD Biosciences), BV421 anti-mouse CD49b (DX5, BD Biosciences), FITC anti-mouse Ly6C (HK1.4, BioLegend), PE anti-mouse F4/80 (C1:A3-1, BD Biosciences), BV605 anti-mouse CD103 (2E7, BioLegend), PerCP/cyanine5.5 anti-mouse CX3CR1 (SA011F11, BioLegend), V500 anti-mouse MHCII (Y3P, BD Biosciences), FVS 780 (BD Biosciences), and corresponding isotype controls. Stained pulmonary immune cells were subjected to flow cytometry analysis using a Fortessa instrument (BD Biosciences). The resulting data were analyzed by FlowJo software (Tree Star, Ashland, OR, USA).

### Mass cytometry analysis

Cell labeling was performed in accordance with the manufacturer's recommendations (Fluidigm, South San Francisco, CA, USA). Briefly, cells were isolated, re-suspended in 0.5 μM Cell-ID cisplatin solution (Fluidigm), and incubated at room temperature for 5 min to stain dead cells. Cells were then washed once with staining buffer (PBS with 0.5% bovine serum albumin and 0.02% NaN_3_) and incubated with mouse, hamster, and rat total IgG (Equitech-Bio, Inc., Kerrville, TX, USA) re-suspended in 50 μL of staining buffer for 20 min on ice to block Fc receptors. To this mixture, 50 μL of an antibody cocktail of surface markers consisting of 38 metal-conjugated antibodies in staining buffer was added. Samples were then incubated on ice for 30 min with gentle agitation. All metal-conjugated antibodies were described in our previous publication 23. After they had been stained, cells were washed twice with 1 mL staining buffer, re-suspended in 0.2 mL fixation solution (0.025% Ir nucleic-acid intercalator in Fix and Perm buffer [Fluidigm Sciences]), and incubated at 4 ℃ overnight. Fixed cells were then rinsed twice with 1 mL Perm buffer (eBioscience Inc., San Diego, CA, USA). An intracellular antibody cocktail consisting of five metal-conjugated antibodies in Perm buffer was added (100 μL) and samples were incubated on ice for 30 min. Cells were then washed once with 1 mL Perm buffer, followed by a single rinse with 1 mL staining buffer. Samples were then washed twice with 1 mL distilled water. Finally, cells were re-suspended in Cell Acquisition Solution-EQ Bead mixture (Fluidigm) at a concentration of 1 × 10^6^ cells/mL, then loaded onto a Helios CyTOF system (Fluidigm) at a rate of ≤ 500 events per second.

The resulting mass cytometry data were filtered to exclude dead cells, doublets, and debris. CD45^+^ cell events of interest were then gated using FlowJo (Tree Star). The gated cell events were clustered using the PhenoGraph or X-shift algorithms. Signal intensities for each marker were transformed by cytofAsinh. For visualization, mass cytometry data from each sample were analyzed using t-distributed stochastic neighbor embedding (t-SNE) algorithms in R version 3.6.1 (R Foundation for Statistical Computing, Vienna, Austria). To characterize the ALI-recruited cells specifically involved in MSC therapy, PhenoGraph and t-SNE algorithms were performed to analyze the recruited cells identified in the first analysis (gating strategy, see Figure S1).

### Single-cell RNA sequencing analysis

Pulmonary immune cells were purified by CD45 MicroBeads (Miltenyi Biotec, Germany) and loaded on the Chromium Controller. Following preparation of single-cell cDNA libraries, sequencing was performed on the Illumina platform. Sequencing data were subjected to quality control and filtering (Figure S2) and then analyzed. For clustering of cells by gene expression, principal component analysis was performed and a graph-based clustering algorithm was implemented in Seurat. For visualization, t-SNE was used to reduce dimensionality. Cells that expressed *Cd45* were isolated, and the major cell populations were identified (e.g., T cells, B cells, natural killer [NK] cells, AMs, conventional type 1 DCs, and other myeloid cells) (Figures S3 and S4). Using mass cytometry, specific analysis of recruited cells (i.e., “other myeloid cells”) was undertaken by means of the analysis workflow as described above. The R package Monocle was used to perform pseudotime analysis. Differentially expressed genes were characterized as those with fold-change > 1.5 and P value < 0.05.

### Depletion of pulmonary Ly6C^hi^ and Ly6C^low^ monocytes

For depletion of pulmonary Ly6C^low^ monocytes, LPS and LPS/MSC groups were intravenously administered 200 μL clodronate-containing liposomes (clo-lips) at 24 h prior to LPS administration, then twice at 24-h intervals, and twice at 48-h intervals to ensure continuous depletion. PBS-containing liposomes (PBS-lips, 200 μL) were administered as placebo treatment. A CCR2 antagonist (BMS CCR2 22, R&D Systems, Minneapolis, MN, USA) was used for selective depletion of pulmonary Ly6C^hi^ monocytes. The CCR2 antagonist was dissolved in absolute ethanol and then adjusted with PBS to a concentration of 0.5 μg/μL. LPS and LPS/MSC groups were intraperitoneally administered 100 μg CCR2 antagonist at 24 h prior to LPS administration, then twice at 24-h intervals, and twice at 48-h intervals to ensure continuous depletion. An equal volume of absolute ethanol was administered as placebo treatment.

### Statistical analysis

Dot plots and heatmaps were generated in GraphPad Prism (version 6.0, GraphPad Inc., La Jolla, CA, USA) and the Statistical Package for the Social Sciences (SPSS) (version 19.0, IBM Corp., Armonk, NY, USA) was used for data analysis. Data are presented as means ± standard errors of the mean. Significant differences were determined by Kaplan-Meier analysis and the *t*-test. P values < 0.05 were considered statistically significant.

## Results

### MSCs protect against LPS-induced ALI

**Figure 1:**
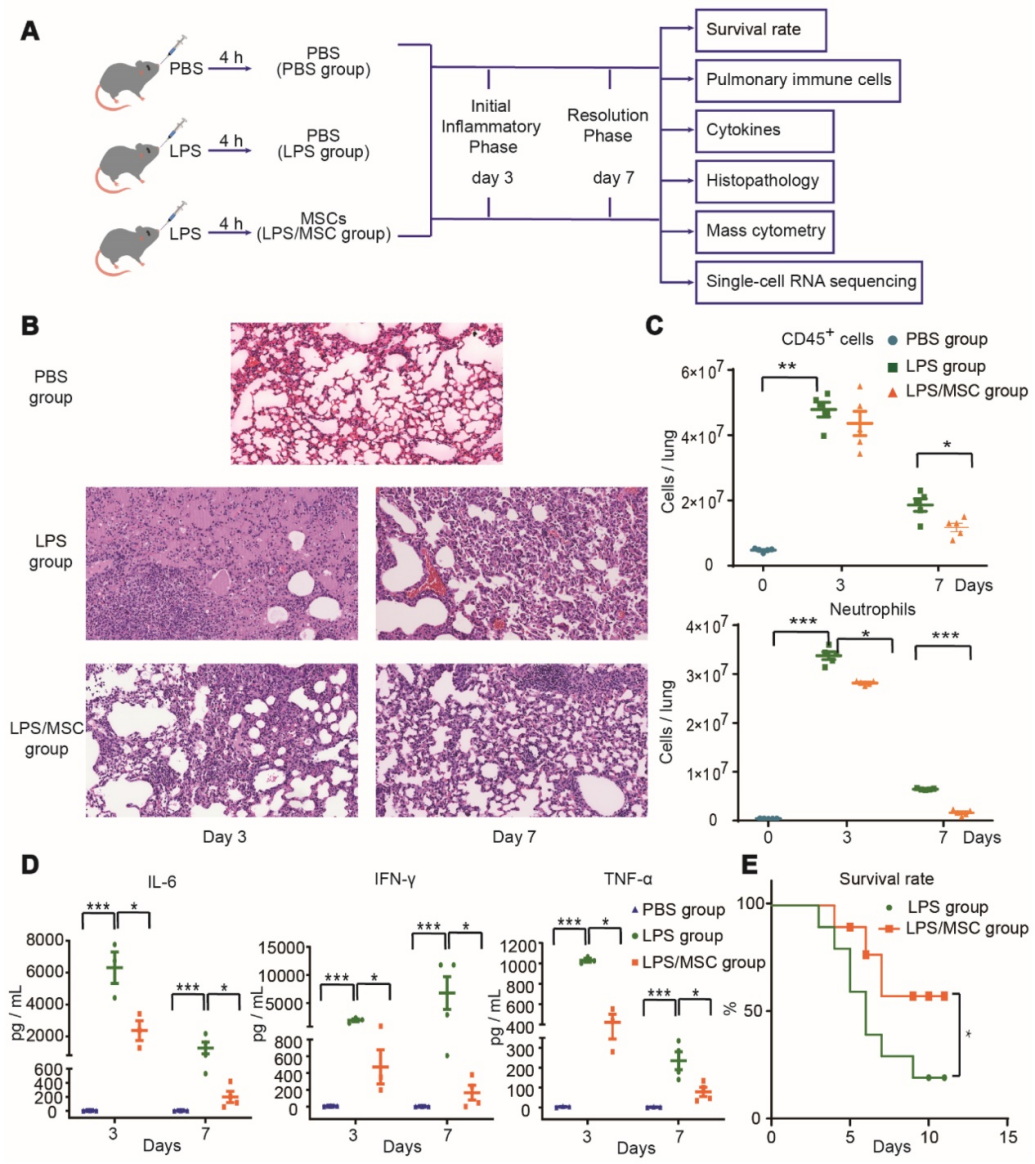
** MSC treatment ameliorated LPS-induced acute lung injury (ALI) in mice. (A)** Experimental protocol for MSC treatment.** (B)** Representative lung histology findings over time after LPS administration or MSC treatment.** (C)** Numbers of CD45^+^ cells and neutrophils in each group over time (n = 5, paired *t*-test). **(D)** Cytokine levels in bronchoalveolar lavage fluid (BALF) in each group over time (n = 3-4, paired *t*-test). **(E)** Effects of MSC treatment on survival rate over time (n = 10, Kaplan-Meier test). * p < 0.05, ** p < 0.01, and *** p < 0.001.

To determine the effect of MSC treatment for ALI, LPS-induced ALI was established in a mouse model by orotracheal administration of LPS. MSCs or PBS were administered orotracheally 4 h later as shown in Figure 1A. Lung tissue sections from three groups were observed under a light microscope, as shown in Figure 1B. The PBS group showed intact and clear alveolar structure, with few inflammatory cells observed in some areas. In the LPS group, the alveolar wall of most areas was broadened with edema, such that the alveolar cavity was collapsed. Furthermore, on day 3 after LPS administration, extensive inflammatory cell infiltration and erythrocyte exudation were observed in the alveolar wall and alveolar cavity. The numbers of CD45^+^ immune cells (especially neutrophils) and the levels of inflammatory cytokines (IL-6, IFN-γ, and TNF-α) in BALF were all significantly increased (Figure 1C-D). After MSC treatment, the pathological lung damage was significantly ameliorated, while inflammatory cytokines in BALF and neutrophils in lung tissue were significantly reduced on days 3 and 7 (Figure 1B-D). Moreover, the survival rate was markedly higher in the LPS/MSC group than in the LPS group (Figure 1E).

### Effect of MSC therapy on monocytes recruited during ALI

The recruitment of lymphocytes and neutrophils has been extensively described in ALI. However, an integrative analysis of changes across all immune populations in the lung during ALI progression and after MSC treatment has not yet been undertaken. To address this gap and identify potential therapeutic targets, we used the Helios CyTOF system to phenotype immune cells thoroughly in the lungs of LPS and LPS/MSC groups over time.

**Figure 2:**
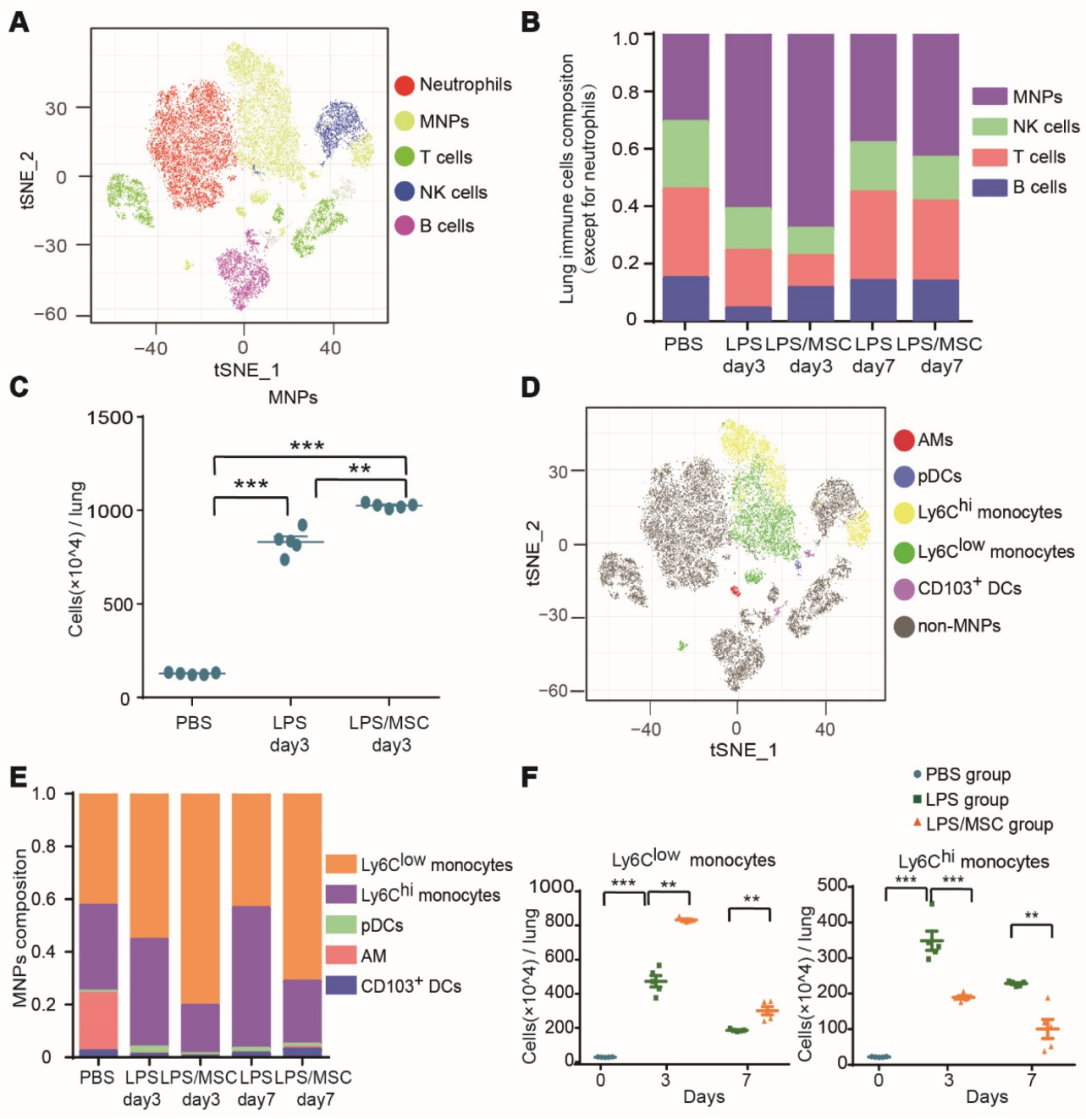
** Mass cytometry identified ALI-recruited immune cell subsets involved in MSC treatment. (A)** viSNE map showing major immune cell subsets in lung tissue.** (B)** Composition of CD45^+^ immune cells (except for neutrophils) in each group over time.** (C)** Numbers of mononuclear phagocytes (MNPs) in each group on day 3.** (D)** viSNE map showing lung myeloid cell subsets. **(E)** Composition of MNPs in each group over time. **(F)** Numbers of Ly6C^hi^ and Ly6C^low^ monocytes in each group over time (n = 5, paired *t*-test). ** p < 0.01 and *** p < 0.001.

Multi-dimensional data were acquired by time-of-flight mass cytometry and analyzed with the t-SNE algorithm. Lung CD45^+^ immune cells for analysis were identified by a sequence of gating steps (Figure S5). The t-SNE analysis was performed on equal numbers of events per sample and data were pooled to generate final two-dimensional density plots (Figure 2A). In visualization of t-distributed stochastic neighbor embedding (viSNE) plots, immune cells were clustered based on their phenotypic similarity. The immune lineages of clusters were determined based on their distinct expression patterns of surface and intracellular markers. This unbiased analysis identified five major populations in lung parenchyma: T cells, B cells, NK cells, neutrophils, and MNPs (Figures 2A and S6A).

The proportions of cell types varied among treatments (Figure 2B). MNPs were prevalent in ALI lung immune compartments, while T cells, B cells, and NK cells were present at lower proportions than those observed in the PBS group. On day 3 after LPS administration, the proportion of MNPs was increased by 61.1% in the LPS group, nearly twice the proportion observed in the PBS group. This proportion was highest in the LPS/MSC group (68%). Similar trends were observed in the numbers of MNPs (Figure 2C). Moreover, on day 7 after LPS treatment, the proportions of MNPs were reduced in both LPS and LPS/MSC groups (38.1% and 43.2%, respectively), although they remained higher than in the PBS group (30.8%) (Figure 2B). These data indicated MNP recruitment into injured lungs and their potential contribution to injured lung repair during MSC therapy, consistent with the findings in previous reports 25, 26.

To explore the recruited MNP subsets during MSC immunotherapy for ALI, pDCs, AMs, CD103^+^ DCs, Ly6C^hi^ monocytes, and Ly6C^low^ monocytes were classified on the basis of the differential expression of CD11b, BST2, Siglec-F, CD103, Ly6C, and CD11c (Figures 2D and S6B). AMs, Ly6C^hi^ monocytes, and Ly6C^low^ monocytes were the three main populations in the lung MNPs of the PBS control group (21.7%, 32.6%, and 42.6%, respectively). The proportions of Ly6C^hi^ and Ly6C^low^ monocytes in lung tissue in the LPS group changed with the progression of inflammation. From day 3 to day 7 after LPS administration, the proportion of Ly6C^hi^ monocytes increased from 40.9% to 53.3%, while the proportion of Ly6C^low^ monocytes decreased from 55.6% to 43.6%. Compared with the LPS group, MSC immunotherapy greatly affected the proportions of Ly6C^hi^ and Ly6C^low^ monocytes. The proportion of Ly6C^hi^ monocytes in the LPS/MSC group increased from 18.4% to 23.9%, while the proportion of Ly6C^low^ monocytes decreased from 80.6% to 71.4% (Figure 2E). The numbers of these cell types exhibited tendencies similar to their corresponding proportions (Figure 2F). Thus, both Ly6C^hi^ and Ly6C^low^ monocytes were recruited into the lungs during ALI, but played a distinct role during MSC treatment of ALI.

**Figure 3:**
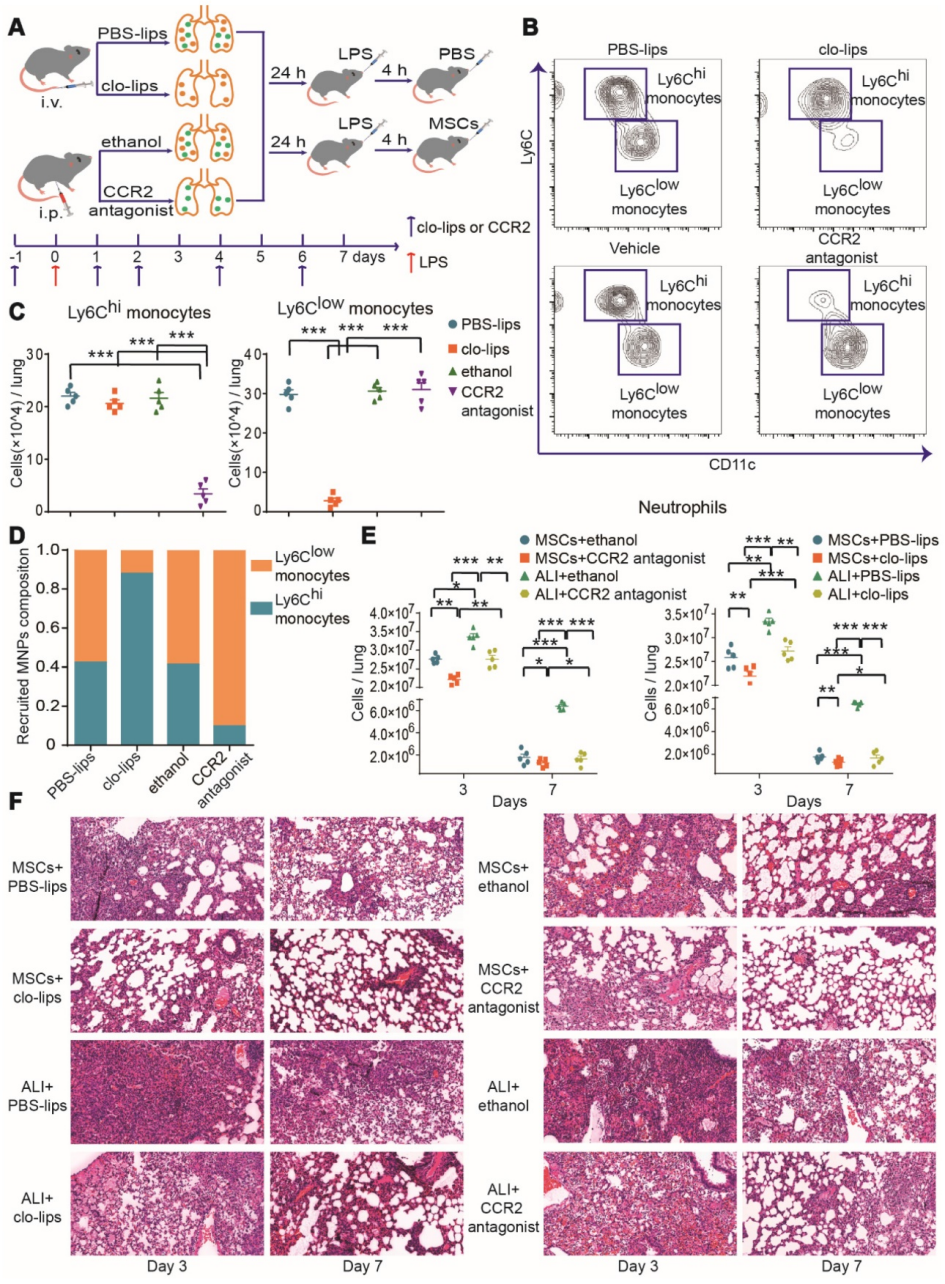
** MSC treatment efficacy in ALI was improved by depletion of Ly6C^hi^ or Ly6C^low^ monocytes. (A)** Experimental protocol for Ly6C^hi^ or Ly6C^low^ monocyte depletion.** (B)** Representative flow cytometry plots showing effects of Ly6C^hi^ or Ly6C^low^ monocyte depletion in the PBS group.** (C)** Effects of clo-lips or CCR2 antagonist treatment on numbers of Ly6C^hi^ and Ly6C^low^ monocytes in the PBS group, determined by flow cytometry. **(D)** Effects of clo-lips or CCR2 antagonist treatment on proportions of Ly6C^hi^ and Ly6C^low^ monocytes in the PBS group, determined by flow cytometry. **(E)** Number of neutrophils in ALI and MSC-treated ALI lungs after Ly6C^hi^ and Ly6C^low^ monocyte depletion, determined by flow cytometry. **(F)** Representative histology of ALI and MSC-treated ALI lungs after Ly6C^hi^ and Ly6C^low^ monocyte depletion. n = 5, * p < 0.05, ** p < 0.01, and *** p < 0.001 by paired *t-*test.

To further investigate the effects of Ly6C^hi^ and/or Ly6C^low^ monocytes during MSC treatment of ALI, Ly6C^hi^ or Ly6C^low^ monocytes were selectively depleted with CCR2 antagonist or clo-lips, separately, before LPS-induced ALI, while other cells were unaffected (Figure 3A-D). The pathological damage was attenuated and neutrophils were reduced in the LPS group after Ly6C^hi^ or Ly6C^low^ monocyte depletion (Figure 3E-F), suggesting that Ly6C^hi^ or Ly6C^low^ monocytes contribute to the development of ALI. MSC immunotherapy without Ly6C^low^ or Ly6C^hi^ monocytes also led to a significant reduction in neutrophil count (Figure 3E). Notably, pathological damage was further attenuated in the LPS/MSC group after Ly6C^hi^ or Ly6C^low^ monocyte depletion (Figure 3F). These findings demonstrated that selective depletion of Ly6C^hi^ or Ly6C^low^ monocytes improved the effect of MSCs in ALI.

### High-dimensional identification of heterogeneity of recruited MNPs

Identification of cell populations solely by surface marker expression is inadequate because recruited monocytes can undergo phenotypic differentiation into macrophages or DCs, and several populations shared similar markers. Moreover, cell subsets can acquire or lose functional capacities because of inflammation that occurs during ALI development. In our study, the MNP population was considered to include recruited monocytes and monocyte-derived cells (Table 1).

**Figure 4:**
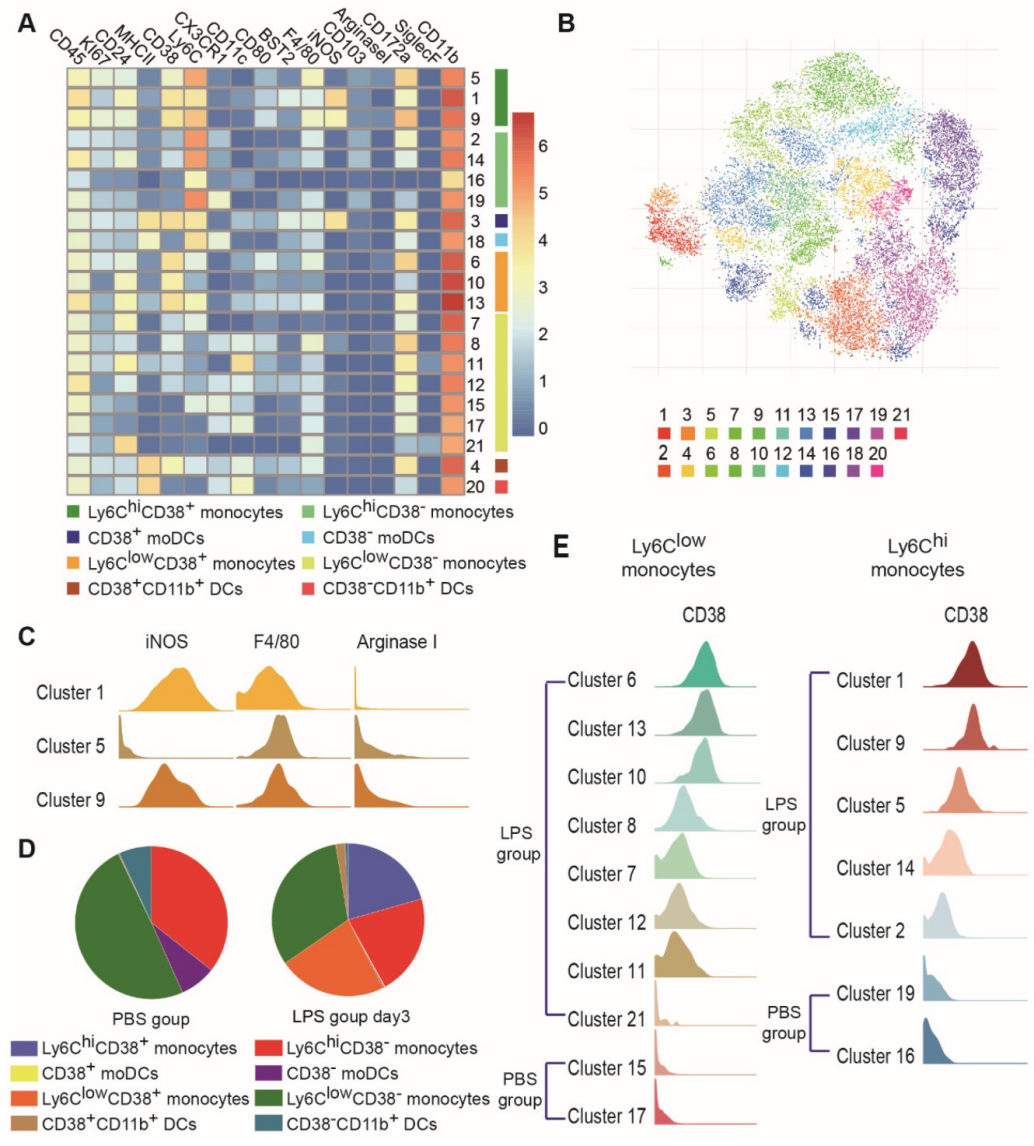
** High-dimensional analysis by mass cytometry revealed heterogeneity among recruited MNPs. (A)** Heatmap showing mean marker expression levels of each cluster in PBS, LPS, and LPS/MSC groups.** (B)** viSNE analysis of recruited MNPs colored according to PhenoGraph cluster in PBS, LPS, and LPS/MSC groups.** (C)** Histograms indicating expression patterns of iNOS, F4/80, and arginase I on clusters 1, 5, and 9 in LPS and LPS/MSC groups. **(D)** Compositions of recruited MNP subsets in PBS and LPS groups.** (E)** Histograms of CD38 expression patterns in Ly6C^hi^ and Ly6C^low^ monocyte subsets in PBS and LPS groups.

Subjective manual analysis of the frequency and marker expression levels of these subsets is complicated and biased. To improve the precision of mass cytometry analysis and probe the immune response induced by MSCs in an unbiased manner, we first excluded B cells, T cells, NK cells, neutrophils, eosinophils, pDCs, AMs and CD103^+^ DCs, and focused on MNPs (Figure S1). We then subjected the mass cytometry data to the PhenoGraph algorithm, combined with the nonlinear dimensionality reduction algorithm (t-SNE). The expression profiles of the recruited MNP clusters were visualized in a heatmap. Marker heterogeneity was assessed at the single-cell level using t-SNE (Figures 4A-B and S6C). This approach led to the identification of nine Ly6C^hi^ MNP phenotypes (clusters 1, 2, 3, 5, 9, 14, 16, 18, and 19) and twelve Ly6C^low^ MNP phenotypes (clusters 4, 6, 7, 8, 10, 11, 12, 13, 15, 17, 20, and 21). Ly6C^hi^ and Ly6C^low^ MNPs were further divided into four subsets based on the level of MHCII expression: Ly6C^hi^ monocytes (clusters 1, 2, 5, 9, 14, 16, and 19), Ly6C^low^ monocytes (clusters 6, 7, 8, 10, 11, 12, 13, 15, 17, and 21), Ly6C^+^MHCII^+^ DCs (clusters 3 and 18; these resembled mo-DCs), and CD11b^+^ DCs (Ly6C^low^CD11c^+^MHCII^+^; clusters 4 and 20) (Table 1).

CD38 is a transmembrane glycoprotein with ectoenzymatic activity, which also functions as a receptor and adhesion molecule 27. CD38-positive cells have been found in the mouse model of ALI, but there have been few analyses of their differentiation and function during the progression of MSC therapy. In this study, cells from the four subsets described above (Ly6C^hi^ monocytes, Ly6C^low^ monocytes, Ly6C^+^MHCII^+^ DCs, and CD11b^+^ DCs) were divided into two groups, based on CD38 expression (i.e., CD38^+^ and CD38^-^) (Figures 4A and S6C). These two groups of cells changed consistently during the progression of ALI. Most clusters of the same cell populations had similar phenotypes; however, subsets of Ly6C^hi^CD38^+^ monocytes differed from each other. Ly6C^hi^CD38^+^ monocytes included clusters 1, 5, and 9. iNOS expression was high in clusters 1 and 9, but almost absent from cluster 5 (Figure 4C). Importantly, high iNOS expression has been associated with a proinflammatory M1 macrophage phenotype.

**Figure 5:**
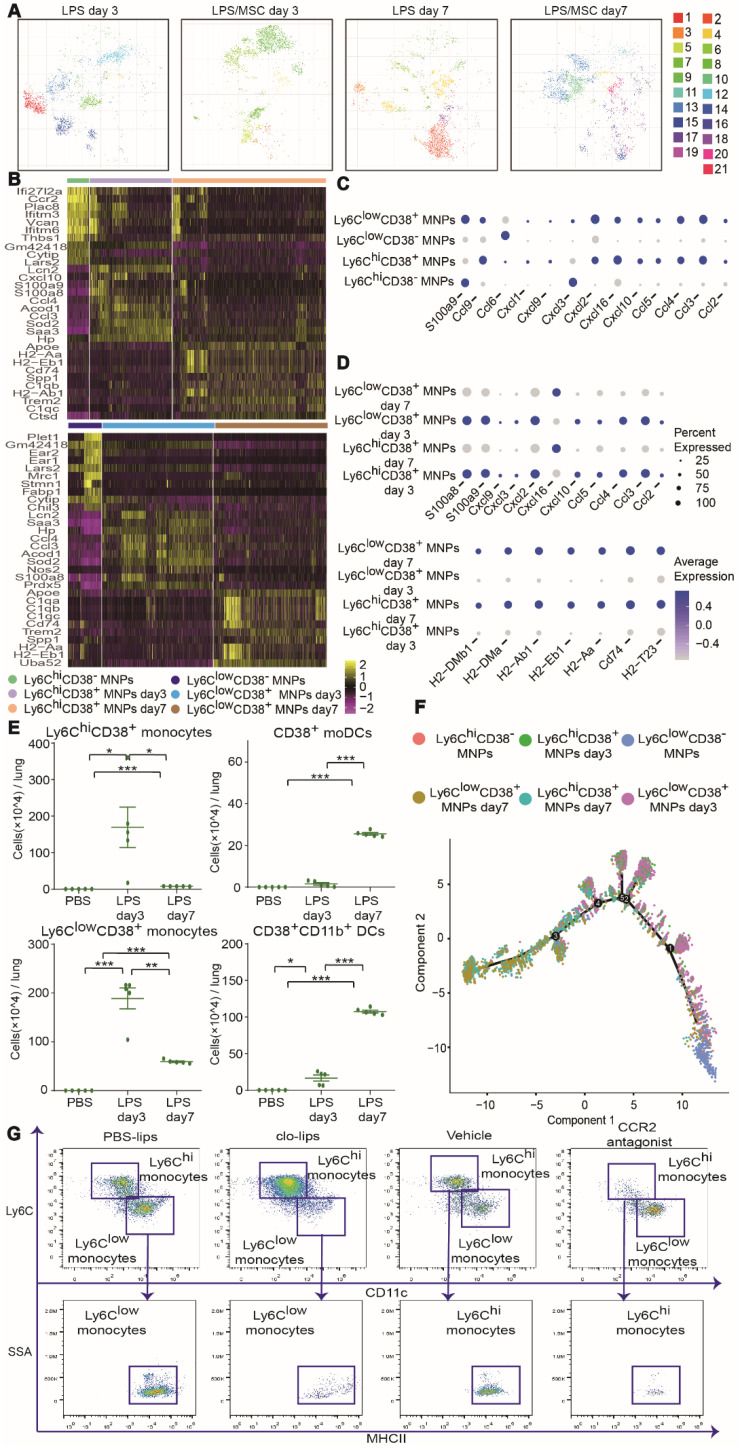
** High-dimensional analysis by mass cytometry and scRNA-seq identified ALI-specific alterations in recruited MNP subsets. (A)** viSNE map showing recruited MNP subsets in LPS and LPS/MSC groups on days 3 and 7, determined by mass cytometry. **(B)** Heatmap of top 10 signature RNA transcripts differentially expressed in Ly6C^hi^ and Ly6C^low^ MNPs in PBS and LPS groups on days 3 and 7, determined by scRNA-seq. **(C)** Bubble heatmap showing genes related to chemotaxis in PBS and LPS groups on day 3, determined by scRNA-seq. **(D)** Bubble heatmap showing genes related to chemotaxis and antigen presentation in PBS and LPS groups on day 7, determined by scRNA-seq. **(E)** Numbers of Ly6C^hi^CD38^+^ monocytes, Ly6C^low^CD38^+^ monocytes, CD38^+^ mo-DCs, and CD38^+^CD11b^+^ DCs in PBS and LPS groups, determined by mass cytometry (n = 5, * p < 0.05, ** p < 0.01, and *** p < 0.001 by paired *t-*test). **(F)** Pseudotime ordering of Ly6C^hi^ or Ly6C^low^ MNPs in PBS and LPS groups over time, determined by scRNA-seq. **(G)** Representative flow cytometry plots showing effects of Ly6C^hi^ or Ly6C^low^ monocyte depletion on mo-DCs and CD11b^+^ DCs.

CD38 expression was absent from clusters of Ly6C^hi^ and Ly6C^low^ monocytes in the PBS group, but increased gradually in clusters from the LPS group (Figure 4D-E). This represented a continuum of recruited monocyte activation. Because lung MNPs displayed distinct cellular signatures in the LPS group and LPS/MSC group over time (Figure 5A), we next mapped the distributions and marker expression patterns of each cluster to distinguish ALI and MSC-specific immune changes.

### Characteristics of recruited MNP subsets during ALI progression

CD38 expression by MNPs can be induced in inflammatory conditions and is associated with an activated phenotype of robust cytokine and chemokine secretion 28. To clarify the characteristics of CD38^+^ MNPs during ALI development, unbiased scRNA-seq was used to compare CD38^-^ MNPs in the PBS group and CD38^+^ MNPs in the LPS group over time.

On day 3 after LPS administration, most genes related to chemotaxis were upregulated in CD38^+^ MNPs (e.g., *Ccl2, Ccl3, Ccl4, Ccl5, Ccl9, Cxcl1, Cxcl2, Cxcl9,* and *Cxcl10*) (Figure 5B-C). On day 7 after LPS administration, the genes for neutrophil chemokines were downregulated, whereas those for antigen presentation were upregulated in CD38^+^ MNPs (e.g., *H2-DMb1, H2-DMa, H2-Ab1, H2-Eb1, H2-Aa,* and* Cd74*) (Figure 5D). These findings imply that, with the onset and progression of ALI, the major roles of Ly6C^hi^CD38^+^ and Ly6C^low^CD38^+^ monocytes changed from chemokine production to antigen presentation. Moreover, the number of mo-DCs and CD11b^+^ DCs were significantly enhanced during ALI progression (Figures 5E and S7A). Recruited monocytes have been reported to differentiate into DCs in the context of ALI 25. Furthermore, our findings suggested that Ly6C^hi^CD38^+^ and Ly6C^low^CD38^+^ monocytes may differentiate into DCs. Notably, mass cytometry data showed that the expression pattern of CD38^+^ mo-DCs (cluster 3) and CD38^+^CD11b^+^ DCs (cluster 4) were similar to those of Ly6C^hi^CD38^+^iNOS^+^ monocytes (cluster 1) and Ly6C^low^CD38^+^ monocytes (cluster 6), respectively (Figure 4A). These results indicated that the elevated proportions of mo-DCs and CD11b^+^ DCs might have been derived from Ly6C^hi^CD38^+^ and Ly6C^low^CD38^+^ monocytes, respectively.

The scRNA-seq and mass cytometry data for a large number of MNPs allowed analysis of the functional states of, and relationships among, Ly6C^low^CD38^+^, Ly6C^low^CD38^-^, Ly6C^hi^CD38^+^, and Ly6C^hi^CD38^-^ cells. We applied the Monocle 2 algorithm to order these subsets in pseudotime, thus characterizing their developmental trajectories. These subsets exhibited a trajectory that began with Ly6C^hi^CD38^-^ and Ly6C^low^CD38^-^ cells, followed by Ly6C^hi^CD38^+^ and Ly6C^low^CD38^+^ cells on day 3, which mainly consisted of inflammatory monocytes. The trajectory ended with Ly6C^hi^CD38^+^ and Ly6C^low^CD38^+^ cells on day 7, which mainly consisted of DCs (Figure 5F). To confirm the results of pseudotime analysis further, we depleted Ly6C^hi^ and Ly6C^low^ monocytes by treatment with a CCR2 antagonist and clo-lips, respectively. CD11b^+^ DCs or mo-DCs were nearly absent after the depletion of Ly6C^low^ or Ly6C^hi^ monocytes, respectively (Figure 5G). Collectively, the above findings suggested that, during the progression of ALI, Ly6C^hi^CD38^+^ and Ly6C^low^CD38^+^ monocytes differentiated into CD38^+^ mo-DCs and CD38^+^CD11b^+^ DCs, respectively. These results are partly consistent with the previous findings where Ly6C^hi^ and Ly6C^low^ monocytes migrated to inflamed tissues followed by differentiation of Ly6C^hi^ monocytes into mo-DCs 29-31, while Ly6C^low^ monocytes differentiated into CD11b^+^ DCs, and both were involved in tissue repair 32.

### Characteristics of MSC-specific alterations in recruited MNP subsets

**Figure 6:**
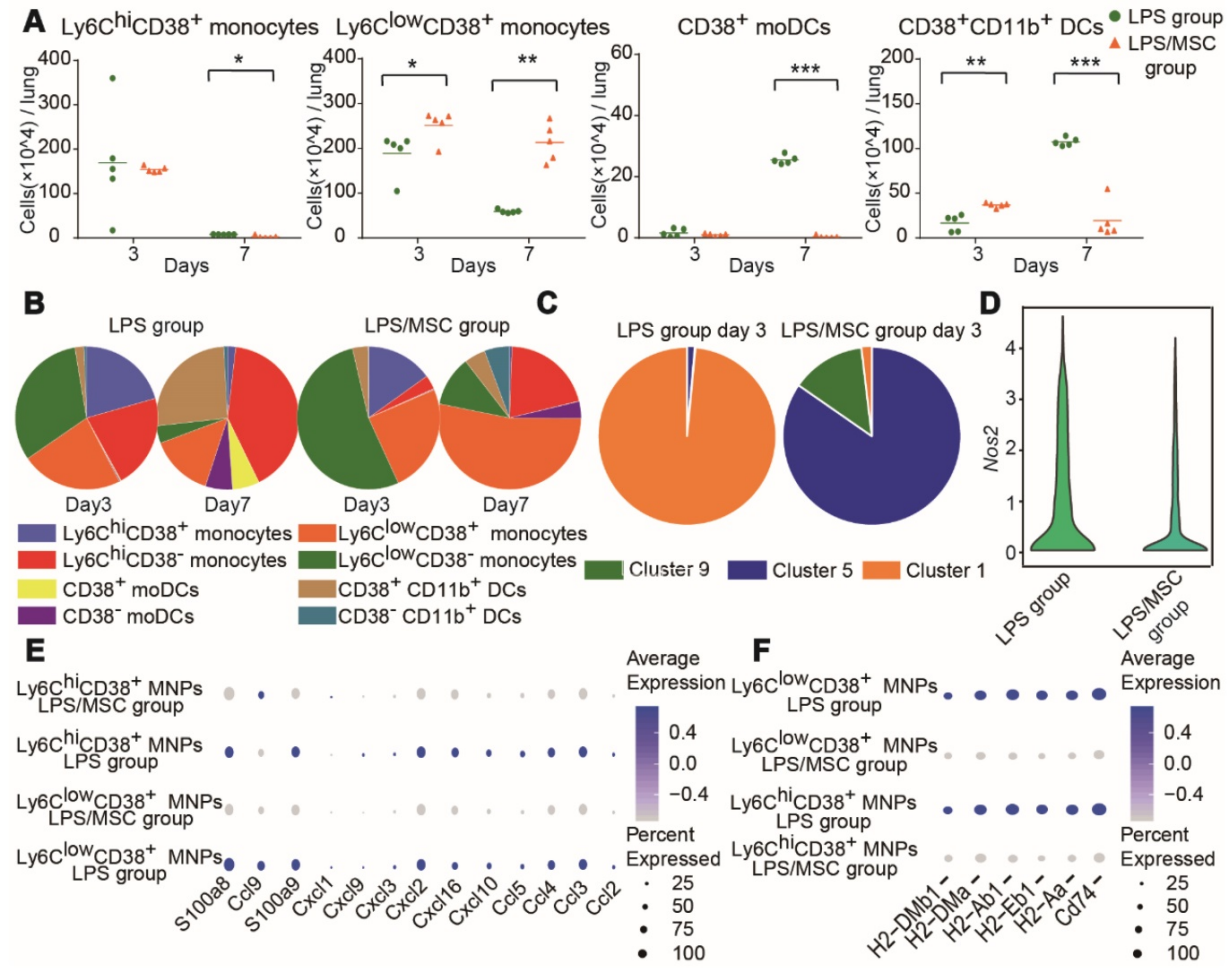
** High-dimensional analysis by mass cytometry and scRNA-seq identified MSC-specific alterations in recruited MNP subsets. (A)** Numbers of CD38^+^ mo-DCs, CD38^+^CD11b^+^ DCs, Ly6C^hi^CD38^+^ monocytes, and Ly6C^low^CD38^+^ monocytes in LPS and LPS/MSC groups, determined by mass cytometry (n = 5, * p < 0.05, ** p < 0.01, and *** p < 0.001 by paired *t-*test). **(B)** Compositions of recruited MNP subsets in LPS and LPS/MSC groups, determined by mass cytometry. **(C)** Compositions of Ly6C^hi^CD38^+^ monocytes in LPS and LPS/MSC groups, determined by mass cytometry. **(D)** Violin plots showing *Nos2* gene expression of Ly6C^hi^CD38^+^ MNPs. **(E)** Bubble heatmap showing genes related to chemotaxis in LPS and LPS/MSC groups on day 3, determined by scRNA-seq. **(F)** Bubble heatmap showing genes related to antigen presentation in LPS and LPS/MSC groups on day 7, determined by scRNA-seq.

Lung MNP subsets in the LPS/MSC group displayed different cellular characteristics compared with the LPS group (Figure 5A). The number and proportion of Ly6C^hi^CD38^+^ monocytes did not significantly differ between the LPS and LPS/MSC groups on day 3 (Figures 6A-B and S7B). However, the most abundant Ly6C^hi^CD38^+^ monocytes were present in cluster 1 in the LPS group, while they were absent from the MSC-treated lung tissues. Furthermore, cluster 5 was mostly observed among Ly6C^hi^CD38^+^ monocytes in the LPS/MSC group, but not in the LPS group (Figure 6C). Data from an earlier portion of this study showed that iNOS expression was higher in cluster 1 than in cluster 5 (Figure 4C). Moreover, the *Nos2* gene and the neutrophil chemokine genes in Ly6C^hi^CD38^+^ MNPs were downregulated in the LPS/MSC group (e.g., *Ccl2, Ccl3, Ccl4, Ccl5, Cxcl2, Cxcl3, Cxcl9,* and *Cxcl10*) (Figure 6D-E). These results suggested that MSC treatment inhibited M1 polarization in Ly6C^hi^CD38^+^ monocytes, and that it reduced production of neutrophil chemokines by Ly6C^hi^CD38^+^ monocytes. The genes for antigen presentation by Ly6C^hi^CD38^+^ MNPs were downregulated in the LPS/MSC group on day 7 (e.g., *H2-DMb1, H2-DMa, H2-Ab1, H2-Eb1, H2-Aa,* and *Cd74*) (Figure 6F). This indicated that MSC treatment inhibited the differentiation of Ly6C^hi^CD38^+^ monocytes into CD38^+^ mo-DCs. Consistent with these results, the numbers of CD38^+^ mo-DCs were significantly reduced in the LPS/MSC group, compared with the LPS group (2.8 × 10^3^ cells/lung vs. 2.55 × 10^5^ cells/lung) (Figure 6A).

Ly6C^low^CD38^+^ monocytes were preferentially enriched in the LPS/MSC group on day 3 (Figure 6A-B), and the expression levels of neutrophil chemoattractant genes were significantly reduced in the LPS/MSC group (e.g., *Ccl2, Ccl3, Ccl4, Ccl5, Ccl9, Cxcl1, Cxcl2, Cxcl3, Cxcl9,* and *Cxcl10*) (Figure 6E). Furthermore, the genes for antigen presentation by Ly6C^low^CD38^+^ cells were downregulated in the LPS/MSC group (e.g., *H2-DMb1, H2-DMa, H2-Ab1, H2-Eb1, H2-Aa,* and *Cd74*) on day 7 (Figure 6F). The numbers of CD38^+^CD11b^+^ DCs were significantly reduced in the LPS/MSC group, compared with the LPS group (1.93 × 10^5^ cells/lung vs. 1.074 × 10^6^ cells/lung) (Figure 6A).

## Discussion

Because of its immunomodulatory effects, MSC treatment has shown substantial efficacy in animal models and preclinical studies of ALI 13. The effect of MSC treatment on the recruitment of MNPs during ALI is widely accepted 14-16. However, multiple MNP subsets coexist in lung tissue and unambiguous identification of recruited MNPs is difficult due to their overlapping and confusing definitions. Although recruited MNPs are important for the development of ALI 25, the roles and characteristics of the recruited MNPs during MSC treatment are not fully characterized. Here, a mouse model of LPS-induced ALI was treated with MSCs to reveal the characteristics of recruited MNPs. Our data indicated that lung injury peaked on day 3 after LPS instillation and that MSC treatment led to improved survival and diminished lung inflammation. MSCs significantly reduced the levels of inflammatory cytokines (IL-6, IFN-γ, and TNF-α) in BALF to promote lung injury repair, which is consistent with the previous finding that blocking TNF-α attenuated the severity of LPS-induced ALI 33. The repair of injured lung tissue consists of two phases: an inflammatory phase that involves lung tissue destruction and pathogen removal, followed by a resolution phase that involves tissue repair. In our study, days 3 and 7 after LPS administration were chosen to represent the inflammatory and resolution phases, respectively.

Based on the gating strategies described by Newell and colleagues 34, the subsets of lung MNPs were studied in detail. These subsets included pDCs, AMs, CD103^+^ DCs, Ly6C^hi^ monocytes, and Ly6C^low^ monocytes. We found that Ly6C^hi^ and Ly6C^low^ monocytes substantially accumulated in the LPS group, while other MNP subsets were under-represented. This indicates that the recruited MNPs during ALI progression are mainly composed of Ly6C^hi^ and Ly6C^low^ monocytes. The different numbers of Ly6C^hi^ and Ly6C^low^ monocytes in the lungs of LPS and LPS/MSC groups imply that both subsets may be involved in MSC treatment of ALI. Selective depletion of Ly6C^hi^ or Ly6C^low^ monocytes improved the efficacy of the MSC therapy in ALI, which further supports our hypothesis.

Our study demonstrated that pulmonary recruited MNPs exhibited phenotypic diversity through a high dimensional analysis. Nemeth et al. proposed that pulmonary recruited MNPs could be removed by injection of clo-lips, and that the repair of lung injury by MSCs mainly depended on enhanced IL-10 secretion from pulmonary recruited MNPs (referred to CD11b^+^ lung cells) co-cultured with MSCs *ex vivo* 17. However, we found that clo-lips administration depleted pulmonary Ly6C^low^ monocytes but not Ly6C^hi^ monocytes, and that CD11b^+^ lung immune cells contained both Ly6C^low^ and Ly6C^hi^ monocytes.

The recruited MNPs exhibited differential expression of CD38, which suggested that they had greater heterogeneity than observed previously. CD38 expression by monocytes and macrophages can be induced during inflammatory processes, such as LPS-induced sepsis and focal ischemia, and has been associated with an activated phenotype and higher levels of secreted cytokines and chemokines 27, 28, 35. CD38 modulates the migration of MNPs toward inflammatory sites by regulating Ca^2+^ mobilization and controlling chemokine receptor signaling 27, 36. We found that CD38-expressing MNPs were absent from naïve lungs, but were significantly increased on day 3 after ALI. Compared with CD38^-^ MNPs in naïve lungs, chemotaxis-related genes were upregulated in CD38^+^ MNPs. These data confirmed the previous report 28 that CD38 expression is related to activated phenotype and maximal chemokine secretion of MNPs. Moreover, previous research showed that CD38^+^ monocytes originate from CD38^-^ monocytes, on the basis of the relationship between the gradual expression of CD38 and the activation of various recruited monocytes 23.

In ALI, Ly6C^hi^CD38^+^ subsets expressed high levels of the M2-macrophage-related gene *Arg1* and the M1-macrophage-related gene *Nos2*. However, only iNOS, a hallmark of M1 macrophage activation, was expressed by Ly6C^hi^CD38^+^ monocytes (Figure S8). Moreover, M2-macrophage-related anti-inflammatory cytokines (e.g., IL-4, IL-10, and IL-13) were almost absent from BALF (data not shown), while M1-macrophage-related proinflammatory cytokines were present at high levels in BALF. These results demonstrated that the expression pattern of Ly6C^hi^CD38^+^ monocytes was more like the M1 phenotype. Macrophage polarization in ALI and subsequent MSC treatment has received increasing attention. Isolated lung macrophages undergo polarization to M2 macrophages and enhance IL-10 secretion to help drive lung repair in the context of MSC treatment 15, 16, 37. Although M1 and M2 macrophages can be clearly distinguished *in vitro*, their genes and phenotypes *in vivo* are more complicated. Therefore, *in vitro* research analyzing the immunomodulatory effect of MSCs on macrophage polarization might not fully mimic the changes *in vivo*.

DCs are regarded as the most potent antigen-presenting cells, governing both T-cell immunity and tolerance. A recent study showed that classical DCs participate in the pathogenesis of inflammatory lung injury by balancing the Th1/Th2 response and regulating cytokine production 38. However, few studies have focused on the comprehensive longitudinal phenotypic and functional plasticity of DCs over time in the context of ALI. In our study, we applied the Monocle 2 algorithm to order recruited MNP subsets in pseudotime and then performed depletion of Ly6C^hi^ and Ly6C^low^ monocytes, respectively. Our findings showed that the CD11b^+^ DCs and mo-DCs were nearly absent after this depletion. Collectively, our results confirmed that mo-DCs and CD11b^+^ DCs were derived from Ly6C^hi^CD38^+^ and Ly6C^low^CD38^+^ monocytes, respectively. Importantly, we found that the numbers of both cells increased with lung repair. Our preliminary experiment also showed that the numbers of T cells were enhanced as the numbers of mo-DCs and CD11b^+^ DCs increased 39. Thus, mo-DCs and CD11b^+^ DCs might regulate the T-cell response in ALI repair.

The various roles of CD38 in monocytes, macrophages, and DCs in ALI lung have been determined 28, while the function of CD38^+^ monocytes in MSC treatment remains unclear. In our study, MSC treatment inhibited the polarization of Ly6C^hi^CD38^+^ monocytes to M1 macrophages during the initial phase of ALI and downregulated the expression levels of neutrophil chemokine genes. These findings implied that MSCs contribute to injured lung repair during the inflammatory phase of ALI mainly by inhibiting M1 polarization and reducing neutrophil chemokine production from Ly6C^hi^CD38^+^ monocytes. Furthermore, MSC immunotherapy downregulated the expression of neutrophil chemoattractant genes by Ly6C^low^CD38^+^ monocytes, but increased the proportion of these cells. This may be related to pathogen clearance, but the LPS-induced ALI model is not suitable for the analysis of pathogen clearance due to its shortage of a self-propagating pathogen, which is the limitation of our study. During the resolution phase (day 7 after LPS administration), MSC treatment inhibited Ly6C^hi^CD38^+^ and Ly6C^low^CD38^+^ monocyte differentiation into CD38^+^ mo-DCs and CD38^+^CD11b^+^ DCs, respectively. Importantly, Ly6C^hi^CD38^+^ monocytes were under-represented in the LPS/MSC group on day 7, suggesting that all cells in this subset differentiated into mo-DCs. However, there were fewer CD38^+^ mo-DCs in the LPS/MSC group compared with the LPS group on day 7. This is presumably due to differentiation into DCs and recruitment of Ly6C^hi^CD38^+^ cells affected by MSC treatment in the LPS/MSC group. Our finding of reduced numbers of CD38^+^ mo-DCs in the LPS/MSC group is also consistent with previous studies that MSCs suppress differentiation of monocytes into DCs thereby suppressing T-cell activation to promote tissue repair indirectly 40-42.

In conclusion, we have characterized the phenotype and functional plasticity of recruited MNPs during ALI progression and uncovered fundamental changes in these cells after MSC treatment. These results have provided a comprehensive longitudinal understanding of the roles of recruited MNPs in MSC-treated ALI and clarified the immunomodulatory effects of MSCs. Our findings should help to pave the way for improving the immunotherapeutic efficacy of MSCs and guide the clinical applications of MSCs in patients with ALI.

## Supplementary Material

Supplementary figures.

## Figures and Tables

**Table 1:** The acute lung injury-recruited mononuclear phagocyte subsets.

Mononuclear phagocytes (MNPs)							
Ly6C^hi^ MNPs				Ly6C^low^ MNPs			
Ly6C^hi^ monocytes		mo-DCs		Ly6C^low^ monocytes		CD11b^+^ DCs	
CD38^+^	CD38^-^	CD38^+^	CD38^-^	CD38^+^	CD38^-^	CD38^+^	CD38^-^
Cluster 1	Cluster 2	Cluster 3	Cluster 18	Cluster 6	Cluster 7	Cluster 4	Cluster 20
Cluster 5	Cluster 14			Cluster 10	Cluster 8		
Cluster 9	Cluster 16			Cluster 13	Cluster 11		
	Cluster 19				Cluster 12		
					Cluster 15		
					Cluster 17		
					Cluster 21		

## Abbreviations

ALI: acute lung injury
AMs: alveolar macrophages
BALF: bronchoalveolar lavage fluid
clo-lips: clodronate-containing liposomes
DCs: dendritic cells
IFN-γ: interferon-gamma
IL: interleukin
LPS: lipopolysaccharide
MNPs: mononuclear phagocytes
mo-DCs: monocyte-derived dendritic cells
MSCs: mesenchymal stem cells
NK: natural killer
PBS: phosphate-buffered saline
PBS-lips: PBS-containing liposomes
pDCs: plasmacytoid dendritic cells
scRNA-seq: single-cell RNA sequencing
TGF-β: transforming growth factor beta
TNF-α: tumor necrosis factor alpha
t-SNE: t-distributed stochastic neighbor embedding
viSNE: visualization of t-distributed stochastic neighbor embedding
