# Supplementary material for: Mesenchymal stem cell-mediated immunomodulation of recruited mononuclear phagocytes during acute lung injury: a high-dimensional analysis study
Source: Theranostics. 2021 Jan 1;11(5):2232–46. doi: 10.7150/thno.52514 (PMC7797670; doi:10.7150/thno.52514)
Supplement: Supplementary file 1 — Supplementary figures. [file thnov11p2232s1.pdf]

Supplemental data

Mesenchymal stem cell-mediated immunomodulation of recruited mononuclear phagocytes during acute lung injury: a high-dimensional analysis study

Jingqi Liu[1†], Pan Li[1,2†], Jiaqi Zhu[1,2], Feiyan Lin[1,2], Jiahang Zhou[1,2], Bing Feng[1,2], Xinyu Sheng[1,2], Xiaowei Shi[1], Qiaoling Pan[1,2], Jiong Yu[1,2], Jianqing Gao[4], Lanjuan Li[1,2], Hongcui Cao*[1,2,3]

1 State Key Laboratory for the Diagnosis and Treatment of Infectious Diseases, Collaborative Innovation Center for Diagnosis and Treatment of Infectious Diseases, The First Affiliated Hospital, Zhejiang University School of Medicine, 79 Qingchun Rd., Hangzhou City 310003, China

2 National Clinical Research Center for Infectious Diseases, 79 Qingchun Rd., Hangzhou City 310003, China

3 Zhejiang Provincial Key Laboratory for Diagnosis and Treatment of Aging and Physic-chemical Injury Diseases, 79 Qingchun Rd, Hangzhou City 310003, China.

4 College of Pharmaceutical Sciences and Dr. Li Dak Sum & Yip Yio Chin Center for Stem Cell and Regenerative Medicine, Zhejiang University, Hangzhou 310058, China

† These authors contributed equally to this work.

***Corresponding author:**

Hongcui Cao

State Key Laboratory for the Diagnosis and Treatment of Infectious Diseases, The First Affiliated Hospital, Zhejiang University School of Medicine, 79 Qingchun Rd., Hangzhou City 310003, China. Tel: 86-571-87236451; Fax: 86-571-87236459

E-mail: hccao@zju.edu.cn

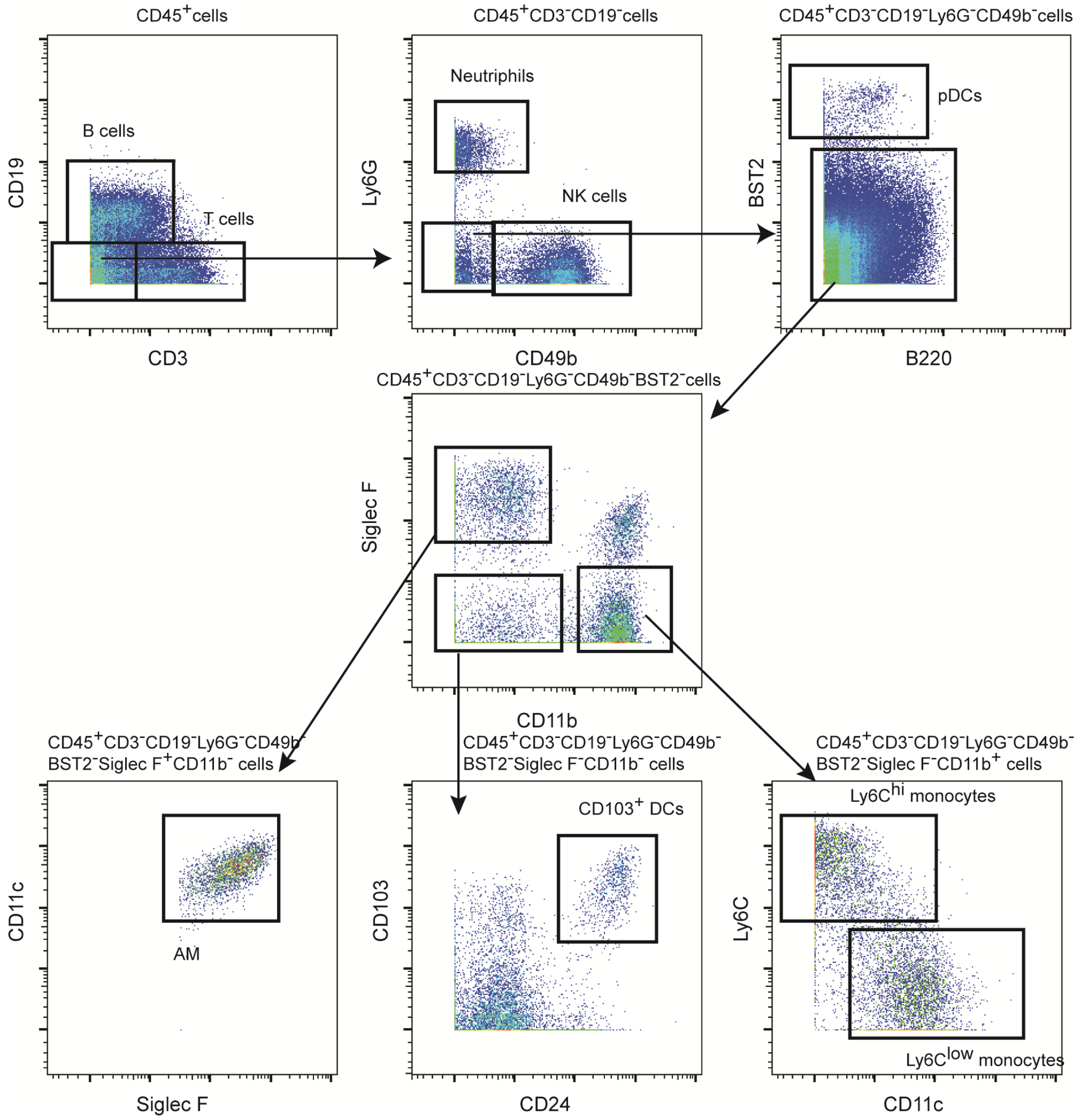

Figure S1. Gating strategy for ALI-recruited MNPs.

A

	PBS	LPS day3	LPS/MSC day3	LPS day7	LPS/MSC day7
Estimated Number of Cells	9380	14283	17235	15058	12958
Number of Reads	375428532	350714839	38590038	388116113	388265679
Valid Barcode	96.9%	97.4%	97.5%	98.1%	98.0%
Sequncing Saturation	56.4%	46.1%	44.1%	48.9%	54.5%
Q30 Bases in Barcode	96.2%	96.3%	96.3%	96.2%	96.3%
Q30 Bases in RNA Read	93.4%	93.4%	93.4%	92.3%	92.9%
Q30 Bases in UMI	95.9%	96.1%	96.1%	96.0%	96.1%
Mean Reads per Cell	40024	24554	22390	25774	29963
Median Genes per Cell	1829	1467	1476	1608	1626
Total Genes Detected	18837	18717	19370	19558	19406
Median UMI Counts per Cell	6434	4634	4415	4722	4542
Reads Mapped Confidently to Intergenic Redions	9.3%	5.9%	5.9%	4.4%	4.0%
Reads Mapped Confidently to Intronic Redions	22.8%	17.5%	17.5%	18.3%	16.1%
Reads Mapped Confidently to Exonic Redions	57.5%	67.9%	69.1%	70.6%	72.9%
Reads Mapped Confidently to Transcriptome	53.0%	63.6%	64.8%	66.8%	69.0%

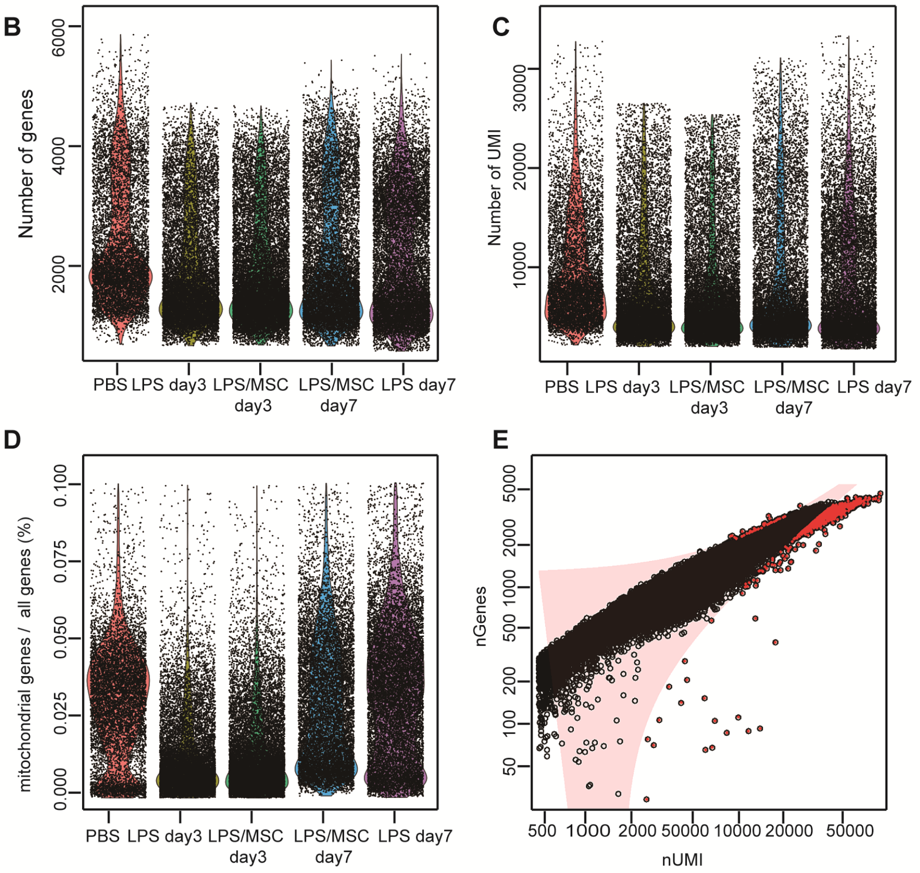

Figure S2. Quality controls for scRNA-seq of mouse lung immune cells. (A) Sequencing parameters of CD45$^+$ mouse lung immune cells subjected to 10×Genomics scRNA-seq platform. **(B)** Genes number of CD45$^+$ mouse lung immune cells within each sample. **(C)** Unique molecular identifier number per cell for each sample. **(D)** Mitochondrial genes/all genes (%) in each CD45$^+$ mouse lung immune cells transcriptomes of each sample. **(E)** Total genes number/cell in relation to unique molecular identifier counts are shown.

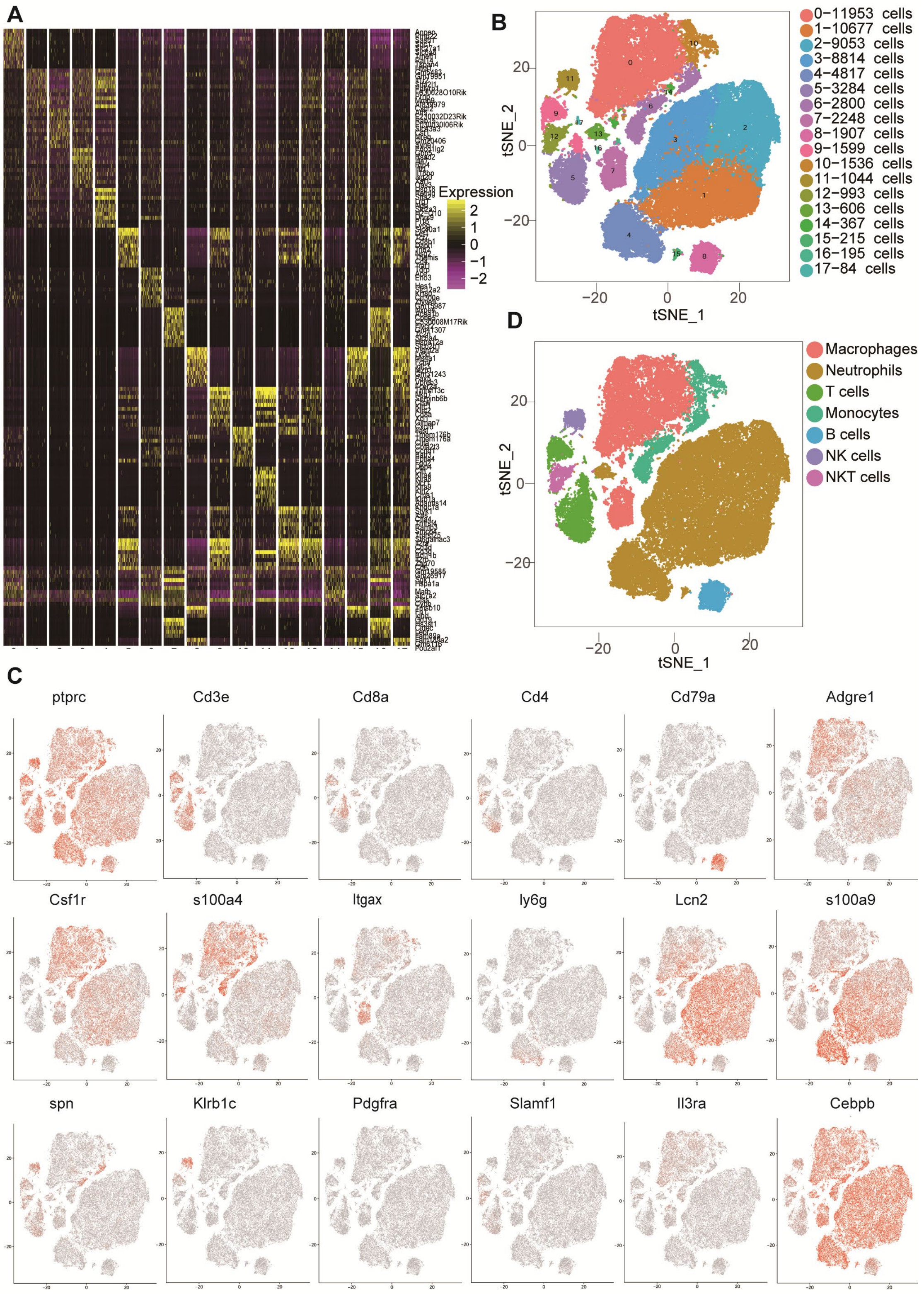

Figure S3. Identification of CD45$^+$ lung immune cell subsets by scRNA-seq. (A) Top 10 signature RNA transcripts differentially expressed in all detected CD45$^+$ mouse lung immune cell clusters. **(B)** 17 clusters across 62192 cells from major immune cell population on viSNE map. **(C)** viSNE map showing marker genes across

major immune cell population. **(D)** viSNE map showing major immune cell subsets in lung tissue.

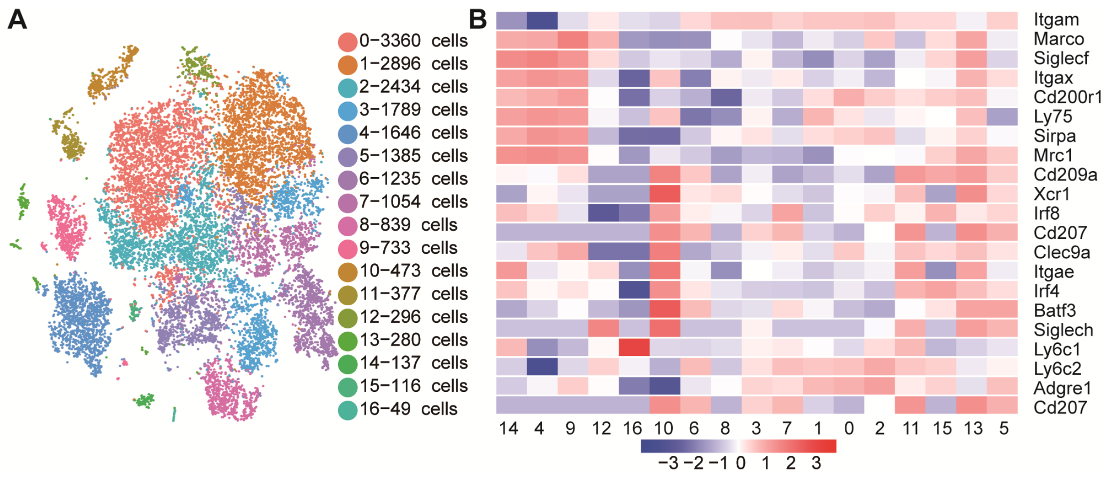

Figure S4. Identification of ALI-recruited MNPs by scRNA-seq. (A) 16 clusters across 19099 cells from lung MNPs subsets on viSNE map. **(B)** Heatmap showing marker genes across lung MNPs subsets.

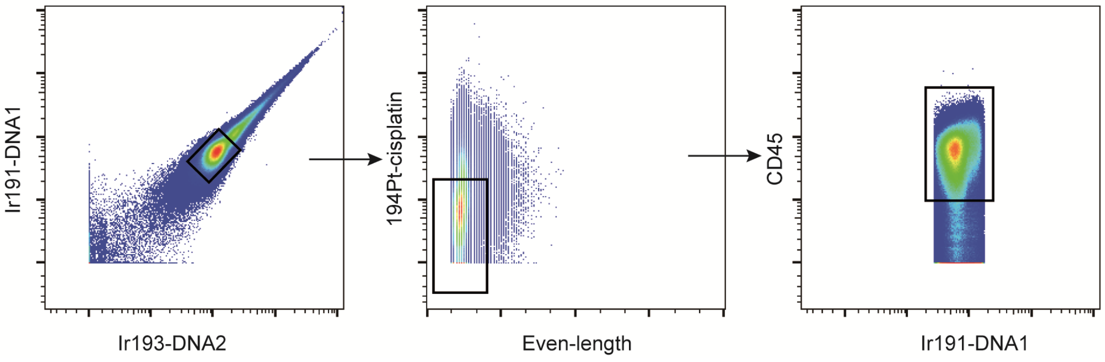

Figure S5. Gating strategy for $CD45^{+}$, single and live cell.

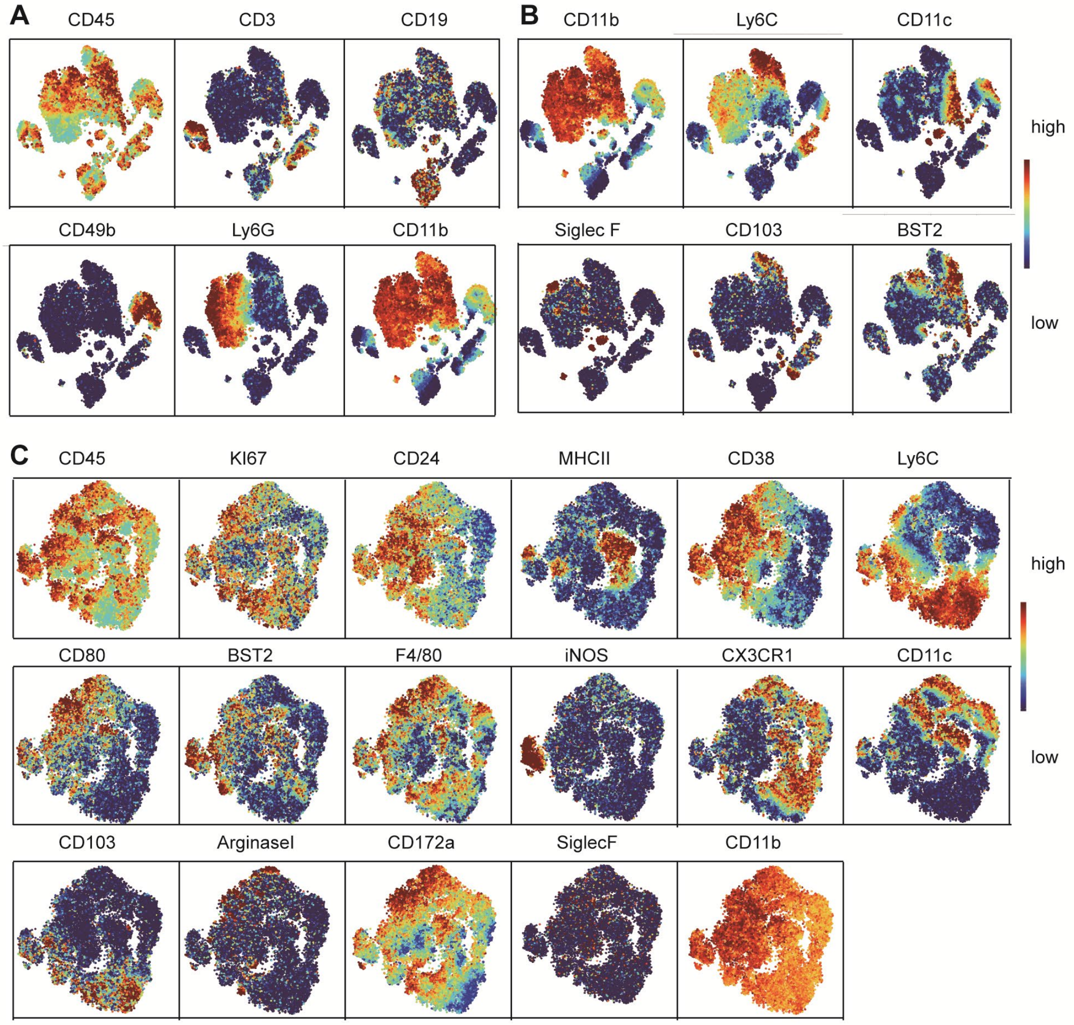

Figure S6. viSNE map showing normalized expression of selected marker. **(A)** Expressed on B cells, T cells, NK cells, MNPs. **(B)** Expressed on MNP subsets. **(C)** Expressed on recruited MNPs subsets.

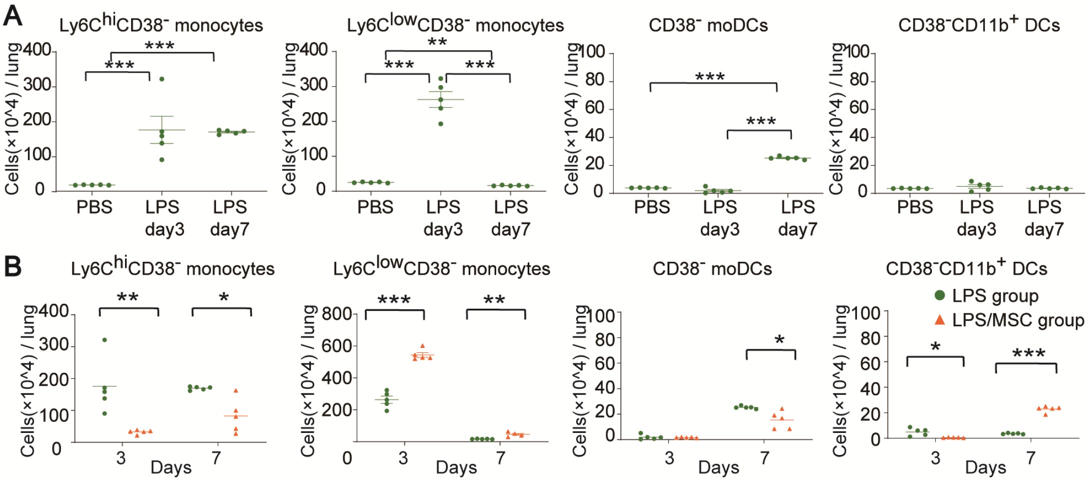

Figure S7. Identification of MSCs-specific alterations in ALI-recruited MNP subsets. (A) Number of Ly6C^{hi}CD38^{-} monocytes, Ly6C^{low}CD38^{-} monocytes, CD38^{-} mo-DCs and CD38^{-}CD11b^{+} DCs in PBS and LPS groups. **(B)** Number of Ly6C^{hi}CD38^{-} monocytes, Ly6C^{low}CD38^{-} monocytes, CD38^{-} mo-DCs and CD38^{-}CD11b^{+} DCs in LPS and LPS/MSC groups. (n=5, **p* < 0.05, ***p* < 0.01, and ****p* < 0.001 by paired *t* test).

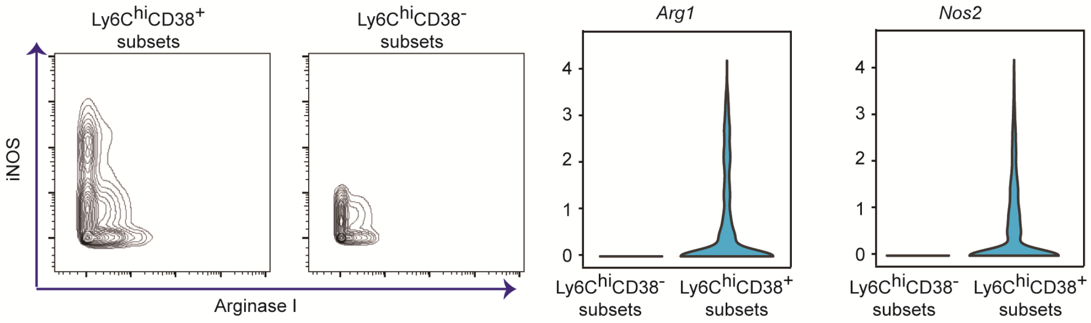

Figure S8. Expression pattern of M1 and M2 specific markers and genes on Ly6C^{hi}CD38^{-} and Ly6C^{hi}CD38^{+} monocytes. (A) iNOS and Arginase I protein expression level on Ly6C^{hi}CD38^{-} and Ly6C^{hi}CD38^{+} monocytes. **(B)** *nos2* and *Arg1* gene expression level on Ly6C^{hi}CD38^{-} and Ly6C^{hi}CD38^{+} monocytes
